# Complete genome sequences of five bacteria isolated from rice plants in a paddy field in Sekinchan, Selangor, Malaysia

**DOI:** 10.1128/mra.00542-24

**Published:** 2024-10-29

**Authors:** Ahmad Zuhairi Abdul Malek, Amalia Mohd Hashim, Nur Fadhilah Khairil Mokhtar, Noor Baity Saidi, Mohd Faizal Abu Bakar, Nallamai Singaram

**Affiliations:** 1 Halal Product Research Institute, Universiti Putra Malaysia, Serdang, Selangor, Malaysia; 2 Department of Microbiology, Faculty of Biotechnology and Biomolecule Sciences, Universiti Putra Malaysia, Serdang, Selangor, Malaysia; 3 Putra Science Park, Office of the Deputy Vice Chancellor (Research and Innovation), Universiti Putra Malaysia, Serdang, Malaysia; 4 Department of Cell and Molecular Biology, Faculty of Biotechnology and Biomolecule Sciences, Universiti Putra Malaysia, Serdang, Malaysia; 5 Malaysian Genome and Vaccine Institute, National Institute of Biotechnology Malaysia, Serdang, Selangor, Malaysia; 6 School of Biosciences, Faculty of Health and Medical Sciences, Taylor’s University Lakeside Campus, Jalan Taylor’s, Subang Jaya, Selangor, Malaysia; The University of Arizona, Tucson, Arizona, USA

**Keywords:** endophytes, long-read sequencing, complete genomes, rice plant

## Abstract

This study examines the genome sequences of five endophytic bacterial isolates from the *Oryza sativa* microbiome to assess their potential as plant bio-inoculants. The five complete bacterial genomes from the genera *Pseudomonas, Burkholderia, Sphingobacterium, Stenotrophomonas,* and *Pantoea* were sequenced using Nanopore long-read sequencing technology.

## ANNOUNCEMENT

Plant-associated bacterial communities such as those in the rhizosphere and phyllosphere play important roles in enhancing plant growth and productivity (1). As part of an ongoing effort to discover bacterial isolates that have biocontrol effects on rice pathogens such as *Xanthomonas oryzae* pv. Oryzae (Xoo) and *Pantoea ananatis* (Pan) and promote plant growth, a culture-dependent approach coupled with *in vitro* characterizations were conducted on bacteria from the rhizosphere and endosphere of *Oryza sativa*. A similar study using endophytes exhibiting antibacterial activity against rice pathogen has also been conducted by another group of researchers (2).

The goal of this study was to identify bacterial isolates that have high potential to be developed as plant bio-inoculants in promoting growth and controlling diseases in rice plants. Root and leaf samples of paddy plants were collected from two paddy fields located in Sekinchan, Selangor (3.4452498, 101.2102732 and 3.4349523, 101.2078464), with permission from the lands’ owner. Three types of samples from the rice plant were used for the bacterial isolation: (1) leaves (2), root, and (3) soil from the rhizosphere region. To obtain samples, leaves were cut into small pieces and ground with a mortar and pestle. For soil samples, roots with soil were immersed in distilled water repeatedly to create a soil solution. Roots were sterilized with 70% ethanol, rinsed with sterile distilled water, and then ground with a mortar and pestle to obtain root samples. The ground leaf, root, and soil samples were diluted tenfold and plated on nutrient agar (NA) and tryptic soy agar (TSA) to grow a variety of bacteria. Five selected bacterial isolates were chosen for genome sequencing based on their positive activity in antimicrobial activity against Xoo and Pan (manuscript in preparation). The selected bacteria were grown in a 20 mL nutrient broth (Oxoid, United Kingdom) for an overnight culture at 35°C. Five milliliters of fresh overnight culture was centrifuged to obtain the pellet, which was then subjected for DNA extraction.

Genomic DNA was extracted using the Qiagen Blood and Tissue DNA Extraction kit (Qiagen, Germany), followed by library preparation using the Ligation Sequencing Kit (LSK-114) (Oxford Nanopore, United Kingdom) and NEBNEXT (New England Biolabs, United Kingdom) protocols. The initial DNA requirement for the library preparation step for all the bacterial DNA is 1,000 ng. Each bacterial isolates’ DNA was sequenced individually using the MinION’s flongle flow cells on the MK1B device until the pores were depleted. Default parameters were used for all softwares, unless otherwise specified. The raw data in the format of .pod5 were subjected to base-calling through Dorado Version 0.5.3 using the model R10 Super Accuracy version 4.3 (https://github.com/nanoporetech/dorado). The Fastq files were filtered for contigs shorter than 2,500 base pairs using Seqtk version 1.3-r106 with the subcommand Seq “-L 2500” for all bacterial genomes, except for Ed8 and Ed1 (https://github.com/lh3/seqtk). The fastq file of Ed8 was preprocessed to remove short contigs and low-quality reads through FiltLong Version 0.2.1 through the parameter of “--min_length 2500 min_mean_q 40 min_window_q 40” (https://github.com/rrwick/Filtlong). Raw sequencing data of Ed1 were not preprocessed due to the low coverage (18X) obtained when assembled using preprocessed data. The preprocessed Fastq files were assembled using Flye version 2.9 (3). Sequence overlapping was detected and trimmed in the Flye assembly’s pipeline. All the genomes were identified as complete circular sequences according to the Flye output. Busco version 5.50 was used to assess the genome qualities using specific models for each genome (4). Taxonomic assignment for each genome was performed using GTDB-tk Version (5). Bakta version 1.92 was used to annotate the genomes (6). The sequencing and annotation summary is presented in Table 1.

**TABLE 1:** Strain sequencing and annotation summary

Strain name	Rh2	Ed8	R2	Ep11b	Ed1
Species^a^	*Pseudomonas taiwanensis*	*Burkholderia seminalis*	*Sphingobacterium* spp*.*	*Pantoea* spp*.*	*Stenotrophomonas cyclobalanopsidis*
SRA ID	SRR28748143	SRR28748141	SRR28748142	SRR28748140	SRR28748139
GenBank ID	CP142890	CP142885, CP142886, CP142887, CP142888, and CP142889	CP142884	CP160631 –CP160632	CP160633
Sanger 16S	PQ001815	PQ001816	PQ001819	PQ001817	PQ001818
Number of reads^b^	10,507	112,018	47,499	28,750	288,911
Average length	10,746.6	8,722.5	8,104.8	8,421.2	14,748
N50	16,780	13,578	11,563	12,770	820
No. of contigs	1	5	1	2	1
Average coverage^c^	21 x	110 x	50 x	60 x	58 x
No of plasmid	0	2	0	1	0
Genome assembly size (bp)	5,374,642	8,481,333	5,354,636	4,033,712	4,233,650
No. of CDS	4,812	7,638	4,503	3,642	3,716
GC content (%)	62.0	66.5	40.0	57.1	66.7
No. of hypothetical genes	454	1,032	909	316	584
No. of rRNAs	22	18	21	22	13
No. of tRNAs	79	72	85	78	76
Busco completion score (%)	99.3	99.9	99.3	99.6	99.4
ANI to the closest genome (%)	99.69	99.09	98.17	99.5	95.58
fastANI reference	GCF_000425785.1	GCF_902832885.1	GCA_002500745.1	GCF_003236715.1	GCF_008710035.1

^a^ Species assignment is predicted via the GTDB-TK tool.

^b^ Total number of reads reported after filtering short sequences below than 2.5 kb, except for Ed1.

^c^ Coverage obtained from Flye assembly output.

Strain Rh2 is identified as *Pseudomonas taiwanensis,* and strain Ed8 is identified as *Burkholderia seminalis* with multipartite chromosomes and three plasmids. Strain R2 is identified as *Sphingobacterium* without a specific species designation. The closest hit of this genome is metagenome assembled genomes (MAG) from a rice plant study (7). Strain Ep11b is identified as the *Pantoea* genus with no proposed species name, with closest hits to a genome isolated from a rice plant in Africa (Bioproject: PRJNA399065). Lastly, strain Ed1 is identified as *Stenotrophomonas cyclobalanopsidis*. These complete genomes will be invaluable resources for further research and development in microbial applications.

## Data Availability

The complete genomes have been deposited in the NCBI under Bioproject PRJNA1056497. The SRAs can be found at SRR28748143, SRR28748140, SRR28748141, SRR28748142, and SRR28748139.
